# Presentation

**DOI:** 10.1590/1516-3180.2019.137080519ap

**Published:** 2019-09-05

**Authors:** Silvia Lisboa, Allison Goldberg, Courtney Burks

**Affiliations:** I Coordinator “Movimento Paulista de Segurança no Trânsito”; II The AB InBev Foundation. Sponsor of the Brasília City Pilot Program

Noncommunicable diseases (NCDs) represent the most significant health challenge of our time and the World Health Organization (WHO) has actively called for a global strategy to mitigate this problem and the risks factors associated with it. The following article describes two Brazilian initiatives directly inspired by the WHO guidelines: the “Movimento Paulista de Segurança no Trânsito” and the “Brasília Vida Segura”. The harmful consumption of alcohol and its effects is a shared concern of both initiatives that could be described as private-public partnerships aiming to mobilize available resources to enhance multidimensional public policies and programs designed to improve the governance, prevention, management and surveillance of NCDs. The management of this complex issue in a context of severe budgetary restrictions requires a financial commitment by local authorities often above available resources. The challenge ahead of the leaders, therefore, concerns the identification of new policies and procedures of engagement between public and private investors to ensure the economic feasibility and sustainability of interventions. In this way, government and civil society, the private sector, the scientific community, schools and trade associations proved to be capable of working together promoting results-oriented public policy and behavior change in an innovative way. Although different in scope, the programs have in common the idea that the solutions to NCDs and their risks factors should be based on scientific evidence, sustainability and feasibility, generating constructive and long term impact in citizens’ lives. The analysis presented by Professors Piquet Carneiro and Rizzo Battistella also revealed the presence of the active engagement of public officials across different areas and the participation of non-governmental agents as a key success factor to the private-public partnership.

The article analyzes public-private partnerships that brought a strategic planning and implementation processes to areas such as road safety, underage drinking and the prevention of harmful alcohol consumption, spreading a collaborative culture and promoting collaborations among stakeholders. The case studies made meaningful use of publicly available data and provided insightful policy recommendations on how these experiments could be replicated.

